# Effect of comfort-oriented intervention combined with medical sponge positioning cushion on treatment compliance and psychological status of patients undergoing side-lying enema operation: A retrospective study

**DOI:** 10.1097/MD.0000000000041717

**Published:** 2025-04-11

**Authors:** Haiyan Li, Bo Peng

**Affiliations:** a Department of Gastroenterology, Wuhan Third Hospital, Wuhan, Hubei, China.

**Keywords:** comfort-oriented intervention, compliance, medical sponge positioning cushion, psychological status, side-lying enema

## Abstract

This study aims to observe the impact of comfort-oriented intervention combined with medical sponge positioning cushion on treatment compliance and psychological status of patients undergoing side-lying enema operation. A total of 110 patients who underwent retention enema operation in the Department of Gastroenterology of our hospital from January 2023 to December 2023 were selected as the research subjects. They were divided into 2 groups based on different nursing interventions. The control group consisted of 50 cases receiving routine care combined with the intervention of medical sponge positioning cushion, while the comfort group consisted of 60 cases receiving comfort-oriented intervention combined with the intervention of medical sponge positioning cushion. The comparison was made based on the coping Hamilton Depression Scale strategy scores, Hamilton Anxiety Scale (HAMA) scores, (HAMD) scores, compliance, and overall satisfaction. The comfort group had a total of 557 enema sessions, with 512 sessions (91.92%) having a drug retention time ≥ 2 hours. The control group had a total of 510 enema sessions, with 436 sessions (85.49%) having a drug retention time ≥ 2 hours. The proportion of drug retention time ≥ 2 hours after retention enema was higher in the comfort group than in the control group (*P* < .05). After nursing, the coping strategy scores of both groups were compared with those before nursing. After nursing, the HAMA and HAMD scores of both groups were compared with those before nursing. It was found that the scores of both groups were lower than before nursing, and the scores of the comfort group were lower than those of the control group after nursing (*P* < .05). The total compliance rate and overall satisfaction rate in the comfort group were 93.33% and 90.00%, respectively. In contrast, the total compliance rate and overall satisfaction rate in the control group were 80.00% and 76.00%, respectively. The total compliance rate and overall satisfaction rate were higher in the comfort group than in the control group (*P* < .05). Comfort-oriented intervention combined with medical sponge positioning cushion can improve treatment compliance of patients undergoing side-lying enema operation, promote patients to face enema with a positive attitude, prolong drug retention time, and improve satisfaction.

## 1. Introduction

Retention enema is a common medical procedure used extensively in the treatment of digestive system diseases. It involves administering liquid medication into the colon and retaining it for a certain period to exert therapeutic effects.^[1]^ However, patients undergoing retention enema may experience physical discomfort, such as the need to maintain uncomfortable positions for an extended period, which may cause pain or discomfort. Moreover, due to the intimate nature of the enema procedure, patients may feel embarrassed or ashamed, leading to anxiety and depressive emotions. Additionally, some patients may have inadequate understanding of retention enema, including its necessity, process, and potential discomfort, which may result in decreased compliance.^[2,3]^ However, conventional nursing typically focuses on ensuring the technical correctness and efficiency of the enema procedure, such as ensuring the correct proportion and temperature of the medication, maintaining aseptic technique during the procedure, and monitoring the patient’s physiological responses during enema administration. Often, this approach overlooks the psychological and emotional needs of patients, failing to fully consider their comfort and subjective experience during treatment. As a result, patients may feel psychologically neglected, which can impact their acceptance and compliance with treatment.^[4]^

Comfort-oriented nursing, as a more comprehensive nursing strategy, not only focuses on the accuracy of technical operations but also emphasizes enhancing patient comfort and improving their overall treatment experience. Recent studies have emphasized the importance of comfort-oriented nursing interventions in various medical procedures. For example, in the context of gastrointestinal endoscopy, comfort-oriented interventions, such as patient education, environment optimization, and psychological support, have been shown to reduce patient anxiety and improve satisfaction.^[5]^ Similarly, research on comfort-oriented care in cancer treatment has demonstrated its potential to enhance emotional well-being, promote coping strategies, and improve compliance with treatment protocols. By creating a more comfortable, safe, and supportive treatment environment, comfort-oriented nursing helps alleviate patient anxiety and depressive emotions, enhances treatment satisfaction, and consequently improves patient compliance and treatment outcomes.^[6]^ Based on this, the aim of this study is to comprehensively explore the application effectiveness of comfort-oriented intervention in retention enema treatment, aiming to provide deeper insights into enhancing patient treatment experience and outcomes.

## 2. Materials and methods

### 2.1. General data

This study was approved by the Ethics Committee of Wuhan Third Hospital (2024-whth-No.25). The study selected 110 patients who underwent retention enema procedures in the Department of Gastroenterology of our hospital from January 2023 to December 2023 as the research subjects. All patients were aged 18 years and above, with the maximum age being 75 years. They were divided into 2 groups based on different nursing interventions. The control group comprised 50 patients who received routine care combined with the intervention of a medical sponge positioning cushion. The comfort group comprised 60 patients who received comfort-oriented intervention combined with the intervention of a medical sponge positioning cushion. Inclusion criteria: retention enema was performed in the lateral position according to the doctor’s advice during hospitalization; age ≥ 18 years old, no gender restriction; there are enema contraindications; patients can communicate with the nursing staff and cooperate with the medical staff to carry out related operations; the clinical data were intact. Exclusion criteria: unconsciousness or accompanied by mental disorders; in the process of enema, there is a strong demand for termination; pregnant or lactating women; have a history of intestinal surgery. There was no difference in the causes of retention enema and education level between the 2 groups (*P* > .05). Since this study was a retrospective study, the Ethics Committee exempted patients from informed consent. Table 1 shows a comparison of baseline information between the 2 groups.

**Table 1:** Comparison of 2 groups of general data.

General data	Conventional group (n = 50)	Comfortable group (n = 60)	χ^2^/*t* value	*P* value
Sex (n)			0.100	.752
Male	29 (58.00)	33 (55.00)		
Female	21 (42.00)	27 (45.00)		
Age (yr)	54.75 ± 12.36	53.65 ± 15.02	0.414	.680
Body mass index (kg/m^2^)	22.98 ± 2.14	23.04 ± 2.28	0.141	.888
Cause of enema (n)			0.125	.989
Ulcerative colitis	22 (44.00)	28 (46.67)		
Constipation	17 (34.00)	19 (31.67)		
Acute pancreatitis	8 (16.00)	9 (15.00)		
Other	3 (6.00)	4 (6.67)		
Standard of culture (n)			0.322	.851
Junior high school and below	9 (18.00)	13 (21.67)		
Secondary school to junior college	24 (48.00)	26 (43.33)		
Bachelor degree and above	17 (34.00)	21 (35.00)		

### 2.2. Methods

The control group received routine care combined with the intervention of a medical sponge positioning cushion: Pre-operation preparation: this includes inspecting the enema equipment, preparing the enema solution, and adjusting the room temperature to ensure patient comfort. The positioning and shape of the medical sponge positioning cushion are adjusted according to the patient’s body size and comfort needs, ensuring that the patient’s side-lying position is stable and comfortable during the enema procedure. Nursing during operation: close monitoring of the patient’s physiological responses during the enema procedure, such as abdominal discomfort, nausea, or any abnormal symptoms, and providing timely care and treatment as needed. Post-operation care: providing guidance on post-enema care, such as dietary advice, activity recommendations, and any symptoms requiring observation, to ensure the patient’s safe recovery.

The comfort group received comfort-oriented intervention combined with the intervention of a medical sponge positioning cushion: Personalized communication strategy: healthcare providers adopt an attitude of active listening, allowing patients to freely express their concerns and expectations. At the same time, healthcare providers observe non-verbal signals from patients, such as body language and facial expressions, to fully understand the patient’s emotional state. During communication, healthcare providers demonstrate empathy by affirming the patient’s feelings and concerns, conveying understanding and respect for the patient’s emotions. Based on the patient’s needs and understanding ability, relevant information is customized, including the purpose and steps of the procedure, the sensations the patient may experience, and how to effectively cope with these sensations. Psychological preparation and support: providing guided relaxation training, such as deep breathing, progressive muscle relaxation, or guided meditation, to help patients achieve a state of physical and mental relaxation before the enema procedure. Guiding patients in mindfulness meditation, focusing on present moment experiences, to reduce anxiety and worry about future or past events. Environment optimization: adjusting the layout of the treatment space to ensure adequate privacy and comfort, such as using partitions or curtains to enhance the patient’s sense of privacy. Properly adjusting indoor lighting, temperature, and sound, using soft lighting, warm tones, and soothing background music to create a peaceful and comfortable environment. Providing comfortable beds/chairs, medical sponge positioning cushions, and appropriate temperatures to ensure the patient’s physical comfort during waiting and treatment processes. Transparency in procedure: providing detailed process explanations, including the purpose and methods of each step, expected sensations, and how patients can cooperate to optimize treatment effectiveness. Introducing patients to the equipment and materials to be used, explaining their functions and roles to enhance patient understanding and trust. Discussing potential discomfort and side effects in advance, and providing specific coping strategies to help patients feel more prepared and in control.

### 2.3. Observed indexes

The drug retention time after retention enema was compared between the 2 groups.

Coping strategy score^[6]^: consists of 20 items, with the scale divided into 3 types of coping strategies: facing (8 items, scores ranging from 8 to 32), avoidance (7 items, scores ranging from 7 to 28), and submission (5 items, scores ranging from 5 to 20). The coping strategy with the highest score indicates the most probable coping mechanism adopted by the patient when facing problems.

Hamilton Anxiety Scale (HAMA) Score^[7]^ and Hamilton Depression Scale (HAMD) Score^[8]^: the HAMA score consists of 14 items, with a total score ranging from 0 to 56, where higher scores indicate more severe anxiety. The HAMD score consists of 24 items, with a total score ranging from 0 to 96, where higher scores indicate more severe depression.

Enema compliance^[9]^: based on patient behavior, compliance is categorized as very good, fair, or poor. Very good compliance is defined as complete cooperation with the enema procedure without resistance. Fair compliance is defined as some resistance behavior but no removal of the rectal tube. Poor compliance is defined as strong resistance behavior, attempted removal of the rectal tube, and requiring assistance from family members to persuade compliance. Both very good and fair compliance can be included in the total compliance rate.

Satisfaction: a self-designed scoring system by our hospital, including ratings for hospital environment, nursing staff enema technique, etc, with a total score of 100. Higher scores indicate greater patient satisfaction, where scores of ≥90 are considered very satisfied, 80 to 89 are satisfied, 70 to 79 are fair, and <70 are dissatisfied.

### 2.4. Statistical processing

All data were processed using SPSS 26.0 statistical software. First, normality test was performed on the measured data, and Shapiro–Wilk test was used to determine whether the data fit the normal distribution. If the data conform to the normal distribution, the mean ± SD is used, and the independent sample *t* test is used for comparison between groups. If the data did not conform to the normal distribution, the Mann–Whitney *U* test (rank sum test) was used for inter-group comparison. For counting data, frequency (n) and percentage (%) were used, and differences between groups were compared by chi-square test (χ² test). In the Chi-square test, for small sample counts (cells with an expected frequency of <5), the Fisher precision test is used for analysis. The significance level of all analyses was *P* < .05.

## 3. Results

### 3.1. Comparison of the drug retention time after retention enema between the 2 groups

The comfortable group underwent colon irrigation a total of 557 times, among which the drug retention time was ≥2 hours 512 times, accounting for 91.92%. The conventional group underwent colon irrigation a total of 510 times, among which the drug retention time was ≥2 hours 436 times, accounting for 85.49%. The proportion of drug retention time ≥ 2 hours after colon irrigation in the comfortable group was higher than that in the conventional group (*P* < .05; Table 2).

**Table 2:** Comparison of OPN, OPG and other indicators between the 2 groups.

Group	Enema times	Drug retention time ≥ 2 h		Drug retention time < 2 h	
		Times (times)	Percentage (%)	Times (times)	Percentage (%)
Conventional group	510	436	85.49	74	14.51
Comfortable group	557	512	91.92	45	8.08
χ^2^ value		11.111			
*P* value		.001			

### 3.2. Comparison of coping style scores between the 2 groups

Before nursing, the coping style scores of the 2 groups were compared (*P* > .05). After nursing, the coping style scores of both groups were found to be higher than before nursing, while avoidance and submission scores were lower than before nursing. Additionally, the comfortable group had higher coping style scores and lower avoidance and submission scores after nursing compared to the conventional group (*P* < .05; Table 3).

**Table 3:** Comparison of coping style scores between the 2 groups (score, $\bar{X}\pm s$).

Group	Number of cases	Face score		Avoidance score		Yield score	
		Before nursing	After nursing	Before nursing	After nursing	Before nursing	After nursing
Conventional group	50	19.63 ± 2.74	25.02 ± 3.01*	19.55 ± 3.24	13.23 ± 2.05*	13.23 ± 2.85	9.12 ± 2.12*
Comfortable group	60	19.41 ± 3.02	27.47 ± 2.94*	19.71 ± 3.06	10.74 ± 1.84*	12.98 ± 3.02	7.74 ± 1.64*
*t* value		0.397	4.305	0.266	6.709	0.443	3.848
*P* value		.692	0	.791	0	.658	0

* Compared with before nursing, *P* < .05.

### 3.3. Comparison of 2 groups of negative emotion scale

In the conventional group, the HAMA score decreased from 22.12 ± 4.03 before nursing to 16.53 ± 2.98 after nursing (*t* = 4.863, *P* < .05). In the comfortable group, the HAMA score also decreased from 21.94 ± 4.17 before nursing to 14.12 ± 2.21 after nursing (*t* = 6.476, *P* < .05). Both groups showed significant improvement after nursing, indicating that both nursing interventions effectively alleviated the patients’ anxiety. Regarding the HAMD score, the conventional group decreased from 23.23 ± 3.95 before nursing to 15.44 ± 2.14 after nursing (*t* = 6.476, *P* < .05), and the comfortable group decreased from 23.37 ± 4.12 before nursing to 12.84 ± 2.06 after nursing (*t* = 6.476, *P* < .05). Both groups also showed significant improvement in depressive symptoms. Statistical analysis revealed significant emotional improvement in both groups after nursing intervention (*P* < .05). However, no significant differences were observed between the groups before intervention (*P* > .05), indicating that the 2 groups had similar emotional states prior to intervention (Table 4).

**Table 4:** Comparison of 2 groups of negative emotion scale (score, $\bar{X}\pm s$).

Group	Number of cases	HAMA score		HAMD score	
		Before nursing	After nursing	Before nursing	After nursing
Conventional group	50	22.12 ± 4.03	16.53 ± 2.98*	23.23 ± 3.95	15.44 ± 2.14*
Comfortable group	60	21.94 ± 4.17	14.12 ± 2.21*	23.37 ± 4.12	12.84 ± 2.06*
*t* value		0.229	4.863	0.181	6.476
*P* value		.819	.001	.857	.001

HAMA = Hamilton Anxiety Scale, HAMD = Hamilton Depression Scale.

* Compared with before nursing, *P* < .05.

### 3.4. Compliance comparison of enema between 2 groups

In terms of enema compliance, the conventional group (n = 50) had 21 cases (42.00%) rated as very good, 19 cases (38.00%) rated as ordinary, and 10 cases (20.00%) rated as poor, with an overall compliance rate of 80.00%. In contrast, the comfortable group (n = 60) had 34 cases (56.67%) rated as very good, 22 cases (36.67%) rated as ordinary, and 4 cases (6.67%) rated as poor, with a significantly higher compliance rate of 93.33%. Statistical analysis showed that the compliance rate in the comfortable group was significantly higher than in the conventional group (χ²=4.365, *P* = .037; Table 5).

**Table 5:** Compliance comparison of enema between 2 groups.

Group	Number of cases	Very good	Ordinary	Poor	Compliance rate
Conventional group	50	21 (42.00)	19 (38.00)	10 (20.00)	40 (80.00)
Comfortable group	60	34 (56.67)	22 (36.67)	4 (6.67)	56 (93.33)
χ^2^ value					4.365
*P* value					.037

### 3.5. Comparison of satisfaction between 2 groups

In terms of patient satisfaction, the conventional group (n = 50) had 8 cases (16.00%) rated as very satisfactory, 20 cases (40.00%) rated as satisfactory, 10 cases (20.00%) rated as ordinary, and 13 cases (26.00%) rated as dissatisfied, resulting in an overall satisfaction rate of 76.00%. In the comfortable group (n = 60), 18 cases (30.00%) were rated as very satisfactory, 29 cases (48.33%) as satisfactory, 7 cases (11.67%) as ordinary, and 6 cases (10.00%) as dissatisfied, yielding a significantly higher satisfaction rate of 90.00%. Statistical analysis indicated that the comfortable group had significantly higher patient satisfaction compared to the conventional group (χ²=3.906, *P* = .048; Table 6).

**Table 6:** Comparison of satisfaction between 2 groups [n, %].

Group	Number of cases	Very satisfactory	Satisfactory	Ordinary	Dissatisfied	Satisfactory degree
Conventional group	50	8 (16.00)	20 (40.00)	10 (20.00)	13 (26.00)	38 (76.00)
Comfortable group	60	18 (30.00)	29 (48.33)	7 (11.67)	6 (10.00)	54 (90.00)
χ^2^						3.906
*P*						.048

## 4. Discussion

Colon irrigation, as a common clinical procedure, has significant effects on patients’ physical and mental well-being. During the process, patients may experience physical discomfort, psychological stress, and concerns about privacy. The combined impact of these factors may decrease patient compliance with treatment and consequently affect treatment outcomes.^[10]^ In this context, comfort-oriented care, as a patient-centered care model, emphasizes the comprehensive consideration of patients’ physical and psychological needs in medical services, providing holistic, meticulous, and personalized care. This care model aims to alleviate patients’ pain and anxiety by improving their environment, emotions, and interaction with medical staff, thereby enhancing patient satisfaction and treatment compliance.

The results of this study indicated that in the comfort group, the proportion of patients with medication retention time ≥ 2 hours was significantly higher than that in the conventional group. This suggests that comfort-oriented interventions help prolong patients’ medication retention time, possibly due to improved positional comfort and reduced psychological stress. Additionally, after nursing care, patients in the comfort group scored higher on the confronting scale compared to the conventional group, while scoring lower on the avoiding scale and yielding scale compared to the conventional group. This indicates that comfort-oriented interventions help promote patients to adopt more active coping strategies, reducing avoidance and yielding as passive coping mechanisms.

This is because comfort-oriented nursing, through personalized communication strategies involving active listening and expressions of empathy, makes patients feel respected and understood, helping to establish a trust relationship between patients and healthcare providers. When patients feel that their concerns and expectations are taken seriously, they are more likely to actively participate in treatment, reducing psychological barriers and improving treatment compliance.^[11,12]^ Furthermore, relaxation training and mindfulness meditation provided in comfort-oriented care help patients achieve a state of physical and mental relaxation before enema administration, reducing fear and tension associated with the process. This psychological relaxation aids in improving patients’ coping mechanisms, enabling them to adopt a more positive attitude towards treatment.^[13,14]^ Finally, the provision of a comfortable and private environment reduces the potential embarrassment and discomfort that patients may experience during the enema process, promoting their positive acceptance of treatment. Elements such as warm colors, soft lighting, and soothing music in the environment help to lower patients’ anxiety levels, further enhancing their positive attitude towards undergoing treatment.^[15]^

In terms of psychological status, postnursing, the HAMA and HAMD scores of both groups of patients were lower than before nursing, with a more significant reduction observed in the comfort group. This indicates that comfort-oriented interventions effectively alleviate patients’ anxiety and depressive states. This is because comfort-oriented nursing guides patients through relaxation training and mindfulness meditation, helping them achieve a calm state of mind and body before the enema process. This helps alleviate anxiety caused by fear, tension, or worries about the unknown. Additionally, by explaining in detail the purpose and methods of each step of the procedure, patients gain a better understanding and anticipation of the upcoming events, reducing anxiety stemming from uncertainty. Moreover, understanding potential discomfort and coping strategies enhances patients’ sense of control, ultimately alleviating depressive moods.^[16]^

Further analysis of patient compliance and satisfaction reveals that the total compliance rate in the comfort group is significantly higher than that in the conventional group, indicating that comfort-oriented interventions can increase patients’ acceptance and cooperation with the retention enema procedure. The total satisfaction rate in the comfort group is also significantly higher than that in the conventional group, which may be attributed to the improvement of patients’ comfort and psychological well-being through comfort-oriented interventions, thereby enhancing overall satisfaction with the enema process. Specifically, when patients feel comfortable and respected during the treatment process, they are generally more willing to follow medical advice and actively participate in treatment. Comfort-oriented interventions enhance patients’ personal comfort, making them more inclined to complete the treatment process, thus improving treatment compliance.^[17,18]^

On the other hand, comfort-oriented interventions improve patients’ overall experience during the retention enema procedure. This nursing model provides personalized care and optimizes the environment, allowing patients to feel more cared for and comfortable, thereby enhancing overall satisfaction. This positive experience encourages patients to provide positive feedback on the treatment process.^[19,20]^ One of the main limitations of this study is the lack of long-term follow-up data. Because the study design was a short-term retrospective analysis, patients could not be followed for long-term evaluation, thus limiting the observation of the persistence of treatment effects and long-term clinical outcomes. This may affect the full understanding of the long-term effects of therapeutic interventions. Therefore, future studies should consider long-term follow-up to assess the efficacy and safety of interventions over longer time periods.

In summary, combining comfort-oriented interventions with medical foam positioning pads can improve treatment compliance among patients undergoing lateral decubitus position enema procedures, promote patients to face the enema procedure with a positive attitude, prolong medication retention time, and enhance satisfaction.

## Author contributions

**Conceptualization:** Haiyan Li, Bo Peng.

**Data curation:** Haiyan Li, Bo Peng.

**Formal analysis:** Haiyan Li, Bo Peng.

**Investigation:** Haiyan Li, Bo Peng.

**Methodology:** Haiyan Li, Bo Peng.

**Supervision:** Bo Peng.

**Validation:** Bo Peng.

**Writing – original draft:** Haiyan Li.

**Writing – review & editing:** Haiyan Li, Bo Peng.
